# Fibrinolytic and coagulative activities of *Yersinia pestis*

**DOI:** 10.3389/fcimb.2013.00035

**Published:** 2013-07-26

**Authors:** Timo K. Korhonen, Johanna Haiko, Liisa Laakkonen, Hanna M. Järvinen, Benita Westerlund-Wikström

**Affiliations:** ^1^General Microbiology, Department of Biosciences, University of HelsinkiHelsinki, Finland; ^2^Department of Bacteriology and Immunology, Haartman Institute, University of HelsinkiHelsinki, Finland

**Keywords:** coagulation, fibrinolysis, hemostasis, plague, plasminogen, proteolysis, *Yersinia pestis*

## Abstract

The outer membrane protease Pla belongs to the omptin protease family spread by horizontal gene transfer into Gram-negative bacteria that infect animals or plants. Pla has adapted to support the life style of the plague bacterium *Yersinia pestis*. Pla has a β-barrel fold with 10 membrane-spanning β strands and five surface loops, and the barrel surface contains bound lipopolysaccharide (LPS) that is critical for the conformation and the activity of Pla. The biological activity of Pla is influenced by the structure of the surface loops around the active site groove and by temperature-induced LPS modifications. Several of the putative virulence-related functions documented for Pla *in vitro* address control of the human hemostatic system, i.e., coagulation and fibrinolysis. Pla activates human plasminogen to the serine protease plasmin and activates the physiological plasminogen activator urokinase. Pla also inactivates the protease inhibitors alpha-2-antiplasmin and plasminogen activator inhibitor 1 (PAI-1) and prevents the activation of thrombin-activatable fibrinolysis inhibitor (TAFI). These functions enhance uncontrolled fibrinolysis which is thought to improve *Y. pestis* dissemination and survival in the mammalian host, and lowered fibrin(ogen) deposition has indeed been observed in mice infected with Pla-positive *Y. pestis*. However, Pla also inactivates an anticoagulant, the tissue factor (TF) pathway inhibitor, which should increase fibrin formation and clotting. Thus, Pla and *Y. pestis* have complex interactions with the hemostatic system. *Y. pestis* modifies its LPS upon transfer to the mammalian host and we hypothesize that the contrasting biological activities of Pla in coagulation and fibrinolysis are influenced by LPS changes during infection.

## *Yersinia* infections and the hemostatic control

The hemostatic mechanism consists of three main phases: primary hemostasis, in which platelets form a hemostatic plug stabilized by fibrin strands; secondary hemostasis i.e., coagulation cascade, which involves a series of linked proteolytic reactions that result in fibrin formation; and tertiary hemostasis in which several mechanisms counteract coagulation processes and induce fibrinolysis (reviewed by van Gorp et al., 1999). The latter involve circulating inhibitors of blood coagulation, endothelium-bound modulators, as well as endothelium-released activators of plasminogen. The plasminogen activators convert the abundant circulating plasminogen to the serine protease plasmin which degrades fibrin and also has a wealth of functions in tissue remodeling and cell migration (reviewed by Myöhänen and Vaheri, 2004; Schaller and Gerber, 2011).

Systemic bacterial infections are a well-known activator of the coagulation cascade, and the activation results from a complex action of bacterial factors, host cytokines, and plasma proteins (reviewed by van Gorp et al., 1999; Sun, 2006; Semeraro et al., 2012). The clotting system normally minimizes blood loss and also modulates innate immune responses, and physically prevents, through fibrin deposition, the spread of invasive bacteria (Yun et al., 2009). In sepsis caused by Gram negative bacteria, the main route for activation of the coagulation cascade is the tissue factor (TF) pathway triggered as a response to endotoxin and/or inflammatory cytokines. Coagulation is preceded by primary fibrinolysis, where tumor necrosis factor (TNF) mediates a transient increase in the levels of tissue type plasminogen activator (tPA) and urokinase plasminogen activator (uPA). This is followed by an increase in plasminogen activator inhibitor 1 (PAI-1) that indirectly suppresses fibrinolysis and favors coagulation and fibrin deposition. Fibrin formation triggers a secondary activation of fibrinolysis, which is rapidly shut-off by the release of high amounts of PAI-1, leading to a procoagulant state as the net effect (van Gorp et al., 1999). Massive thrombin formation and fibrin deposition in bacterial sepsis thus involve overexpression of inflammatory mediators, the microbe(s) and its derivatives, aberrant expression of TF, impairment of physiological anticoagulant pathways, and suppression of fibrinolysis by PAI-1 (Semeraro et al., 2012). Overwhelming infection can lead to severe unbalance in the system, prompting thrombin and fibrin formation or, in more severe case, disseminated intravascular coagulation (DIC) with formation of microvascular thrombi in various organs. DIC eventually consumes blood-clotting factors and thus subsequently contributes to hemorrhage, so thrombosis and bleeding may both be presenting clinical features (van Gorp et al., 1999).

Infections by species of *Yersinia* alter the hemolytic balance and their pathogenesis is influenced by coagulation and fibrinolysis factors. Fibrinogen/fibrin deposition has been observed in infections by *Yersinia pseudotuberculosis* (Caruso, 1986; Fisher et al., 2007), which is the genetically closest species to *Y. pestis*. Luo et al. (2011) found that fibrinogen-deficient mice show increased hepatic bacterial burden and mortality following i.p. or i.v. inoculation with *Yersinia enterocolitica*. The fibrinogen-deficient mice displayed impaired cytokine and chemokine production and suppressed neutrophil recruitment. Similar outcome and phenotype were observed in mice with low TF activity and in mice deficient for PAI-1 or for thrombin-activatable fibrinolysis inhibitor (TAFI; Luo et al., 2011). These mutations cause fibrin deficiency in mice, and it was concluded that TF, PAI-1, and TAFI have critical roles in host defense against the bacteria. On the other hand, fibrin also facilitates both innate and T cell-mediated defense against *Yersinia pestis* (Luo et al., 2013) *Y. enterocolitica* as well as *Y. pseudotuberculosis* infections mostly manifest as self-limiting enterocolitis, but the bacteria can occasionally also cause sepsis. The highly invasive *Y. pestis* possesses the Pla protease that *in vitro* degrades both PAI-1 and TAFI, as will be discussed below, and is able *in vivo* to overcome fibrin-mediated physical entrapment and inflammatory reactions caused by the bacteria (Degen et al., 2007). A functional homolog of Pla is lacking in *Y. pseudotuberculosis* as well as in *Y. enterocolitica*, and the presence or absence of Pla in these three species has a profound impact on their interactions with the hemolytic system.

The evidence for the role of Pla in the virulence of *Y. pestis* is strong. Transcription analyses have shown that the *pla* gene is expressed in buboes, the lung, the spleen, and the liver of *Y. pestis*-infected mice (Sebbane et al., 2006; Lathem et al., 2007; Liu et al., 2009). Deletion of *pla* increases the LD_50_ value million fold in mice infected subcutaneously (Sodeinde et al., 1992), and in the pneumonic form of plague, proteolytically active Pla promotes bacterial proliferation in the lungs (Lathem et al., 2007). However, Pla is dispensable in primary septicemia plague (Sebbane et al., 2006), where the flea injects bacteria directly into the blood vessels, and the deletion of *pla* does not change the LD_50_ when the mice are infected intravenously or intraperitoneally (Sodeinde et al., 1992). On cellular level, it is known that in bubonic plague Pla enables bacterial dissemination from the skin to the lymph nodes where *Y. pestis* multiplies and causes swollen lymphs, or buboes (Sodeinde et al., 1992). Guinet et al. (2008) observed that bacterial loads of Pla-positive *Y. pestis* became higher than those of wild type (i.e., Pla-deficient) *Y. pseudotuberculosis* in rat lymph nodes at 24–48 h after infection when also significant histopathological changes were evident. They concluded that *Y. pseudotuberculosis* infection induced an organized leukocyte response that was not seen with *Y. pestis*. Thus, the role of Pla in plague seems to be restricted to enhancement of bacterial migration from the intradermal infection site into lymph nodes and to their proliferation in buboes and the lungs.

Secreted proteases targeting human coagulation or fibrinolysis proteins have been described in other pathogenic bacteria as well, e.g., *Pseudomonas aeruginosa, Bacillus anthracis*, and *Staphylococcus aureus* (Beaufort et al., 2008, 2010; Chung et al., 2011). Two main features distinguish Pla from these extracellular proteases. First, Pla is a transmembrane, cell-wall associated protease/adhesin that can cause surface-bound proteolysis to optimally advance cell migration across fibrin deposits and subepithelial basement membranes. This analogy to behavior of metastatic cancer cells led us to the concept of bacterial metastasis (Lähteenmäki et al., 2005a) to underline that migration of prokaryotic and eukaryotic cells across tissue barriers is based on similar principles. By contrast, the enzymes of *P. aeruginosa, B. anthracis*, and *S. aureus* are secreted to cell surroundings where their concentration and activity will be diluted. Second, in cleaving plasminogen, PAI-1, and single chain uPA (scuPA), Pla shows awesome cleavage site specificity not seen with the secreted proteases. Here we will discuss the fibrinolytic and coagulative activities that have been characterized for the Pla protease. Pla also has other proteolytic functions as well as adhesive and invasive activities, and for recent more general reviews on Pla and omptins, the reader is referred to Haiko et al. (2009b) and Caulfield and Lathem (2012).

## Structure-function relationships of Pla

### Structure

**Figure 1:**
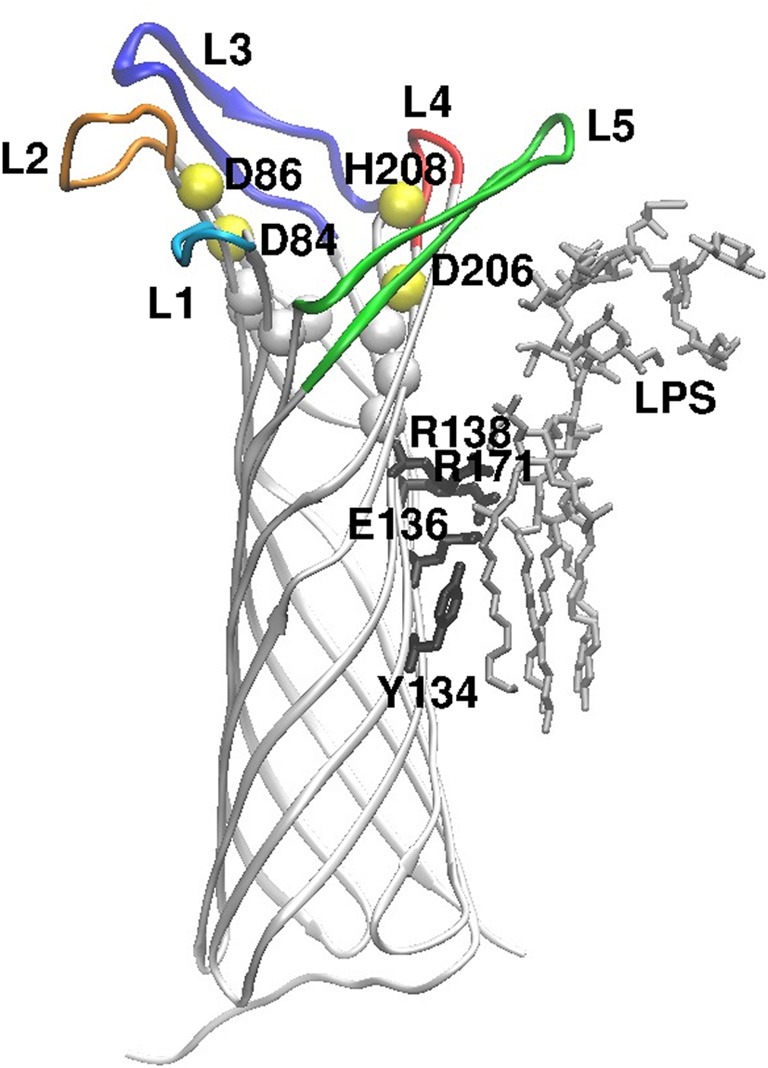
**Overall structure of the Pla protein, oriented perpendicular to the membrane plane, exracellular side up.** The peptide chain is shown as white strands, and the extracellular loops are colored (L1–L5). The yellow spheres show positions of the active site residues (named) and the white spheres locate conserved residues at the bottom of the active site. On the right side of the image, LPS and the amino acids that contact it are shown in stick models (gray and black, respectively). The figure is modified from the crystal structure of Pla (Eren et al., 2010; Eren and van den Berg, 2012).

Omptins are monomeric β-barrel proteins with 10 antiparallel β-strands, and the crystal structures of OmpT of *E. coli* (Vandeputte-Rutten et al., 2001) and Pla have been resolved (Eren et al., 2010; Eren and van den Berg, 2012). The connecting loops on the periplasmic side of the barrel are short (2–4 amino acids) but the five surface-exposed loops (L1–L5) are long and variable in sequence between different omptins. The barrel length is about 70 Å of which 30 Å is located inside the lipid bilayer in the outer membrane, as determined by the two girdles of hydrophobic amino acids (Figure 1).

The catalytic residues are conserved in all omptins (Kramer et al., 2001; Vandeputte-Rutten et al., 2001; Haiko et al., 2009b, 2010) and have been identified in Pla as D84, D86, D206, and H208 (Kukkonen et al., 2001; Eren et al., 2010; Eren and van den Berg, 2012). β-barrel structures are unique to bacterial outer membrane proteins, and the omptin active site residues cross the barrel opening: D84–D86 dyad lying on one side of it and D206–H208 on the opposite side. In earlier work (Eren et al., 2010), a water molecule was seen to connect the two pairs of catalytic residues and was assumed to act as the reactive nucleophile, but in the most recent crystal structure (Eren and van den Berg, 2012) the bound substrate has replaced the water molecule. In addition, S99 and H101 in the vicinity of the active site are important for enzymatic activity of Pla (Kukkonen et al., 2001) since they coordinate the D84–D86 dyad via bridging water molecules (Eren et al., 2010).

### Cleavage specificity

Omptins are endopeptidases that cleave proteins preferentially between basic amino acids (Dekker et al., 2001; McCarter et al., 2004; Hwang et al., 2007; Agarkov et al., 2008). This preference, obtained from quantitative studies with small peptide substrates (2–10 amino acids), does not completely hold for the physiological substrates, where usually only the first position is basic, while the second position varies. Pla cleaves plasminogen between residues R561 and V562, PAI-1 at R346-M347, and scuPA at K148-K149, and Pla also autoprocesses itself between residues K262–N263. In all these target molecules, the cleavage is a single cut of a peptide bond located in an accessible loop structure.

Omptins share high sequence identities and their structures are very similar, but they differ in polypeptide substrate specificity. Detailed analysis of the crystal structures suggests (Eren et al., 2010; Eren and van den Berg, 2012) that minor changes in the Pla barrel dimensions control enzymatic activity. The substitution analyses and chimera constructions have shown that the functional heterogeneity of omptins results from sequence variations in the surface loops that border the barrel opening (Kukkonen et al., 2001; Ramu et al., 2008; Haiko et al., 2011). Thus, the omptin catalytic amino acids are spatially highly conserved, but the specific loop composition is an essential feature that distinguishes Pla functions from those of other omptins and allows correct recognition of large polypeptide substrates.

Omptins have been earlier classified either as serine proteases or aspartate proteases but the more is known about them, the more clearly they seem to form a protease class of their own (Kramer et al., 2001; Vandeputte-Rutten et al., 2001; Eren et al., 2010; Eren and van den Berg, 2012). This consideration stems from their proposed cleavage mechanism, resistance to classical protease inhibitors, neutral pH-optimum, and dependency on bound lipopolysaccharide (LPS). Very recently, we have recorded data that seems to question the well-accepted definition of omptin active site: the Pla mutants D86A, D206A, H208V are inactive in plasminogen activation, as seen before (Kukkonen et al., 2001), but are able to cleave and activate precursor of the human plasminogen activator uPA (Järvinen et al., 2013). Clearly, details of the reaction mechanism of omptins still remain unknown and need further clarification.

### Bound lipopolysaccharide activates Pla

Omptins are unique proteases in that they require rough (short O side chain) LPS to be active (Kramer et al., 2000, 2002; Kukkonen et al., 2001, 2004; Brandenburg et al., 2005; Suomalainen et al., 2010). A consensus protein motif for binding to 4′-phosphate in lipid A (Ferguson et al., 2000) is present in the omptin barrel (Vandeputte-Rutten et al., 2001) and consists of R138 and R171 in Pla. Substitution of these arginines, in particular R138, decreases Pla enzymatic activity and the amount of Pla in cells (Suomalainen et al., 2010). Pla also binds LPS acyl chains via Y134 and E136, and removal of LPS causes subtle conformational changes that lead to inactivation of Pla (Eren et al., 2010). The biggest structural differences between LPS-depleted and LPS-containing Pla are observed in loops L4 and L5, i.e., near the binding site of LPS (Eren and van den Berg, 2012). LPS binding induces narrowing of the active site groove by pushing of the β7-strand inward, which affects the active site geometry. In the absence of LPS, Pla substrate binds deeper in the active site groove and replaces the water molecules essential for the proteolytic mechanism (Eren and van den Berg, 2012). Smooth LPS molecules with long O side chains extend much further out from the bacterial surface than the surface loops of omptins, and thus sterically hinder the loops from recognizing the substrates (Kukkonen et al., 2004). *Y. pestis* lacks genes for O side chain synthesis and therefore has rough LPS and thus active Pla (Skurnik et al., 2000; Prior et al., 2001; Kukkonen et al., 2004). Other bacteria, such as *Salmonella enterica* serovar Typhimurium, modify their LPS structure inside mammalian cells, where the omptin activity is high (Lähteenmäki et al., 2005b).

### Modifications in LPS influence Pla activity

Plague is a zoonotic disease, where the bacterium is transmitted to mammals by a bite of an infected flea; the change of host involves change of temperature from 20–25°C to 37°C. *Y. pestis* modifies its LPS structure upon this temperature shift, and while the LPS is mostly in the tetra-acylated form and the lipid A phosphates are poorly substituted with 4-amino arabinose at 37°C, at 25°C the LPS is mostly hexa-acylated and heavily substituted at lipid A phosphates (Kawahara et al., 2002; Knirel et al., 2005). It is well-established that the *Y. pestis* LPS from 37°C is poorly recognized by Toll-like receptor 4 and hence elicits only poor inflammatory response in mouse and human macrophages, whereas the reverse is true for *Y. pestis* LPS from 25°C (Kawahara et al., 2002; Knirel et al., 2005; Montminy et al., 2006). Thus, the lipid A alteration is essential for evasion of innate immunity responses by *Y. pestis*.

The temperature shift and the LPS alterations also affect the functions of Pla. The activity of Pla in *Y. pestis* is dramatically higher in cells grown at 37°C than in cells grown at 20°C (Suomalainen et al., 2010). The expression of Pla is only modestly up-regulated at 37°C (Motin et al., 2004; Chromy et al., 2005) and the high activity of Pla in cells from 37°C results from temperature-induced LPS alterations. Reactivation of LPS-depleted His_6_-Pla with LPSs from three *Y. pestis* strains gave higher activity with LPSs from 37°C than from 25°C, and analyses with characterized LPS molecules showed that the critical features were (1) the presence of a complete core region in the rough LPS, and (2) low levels of acylation and phosphate substitution in lipid A (Suomalainen et al., 2010), which all characterize the *Y. pestis* LPS in cells grown at 37°C. Exactly how these LPS modifications change the conformation of Pla is not known at the moment. Biology considered, the host shift from the flea to the mammal however represents a huge difference in pathogenic potential: at 20–25°C, i.e., in the flea and at early stages of infection in the mammal host, *Y. pestis* is associated with high innate immune response and low activity of Pla, whereas at 37°C the situation is reversed.

## Pla enhances fibrinolysis

**Figure 2:**
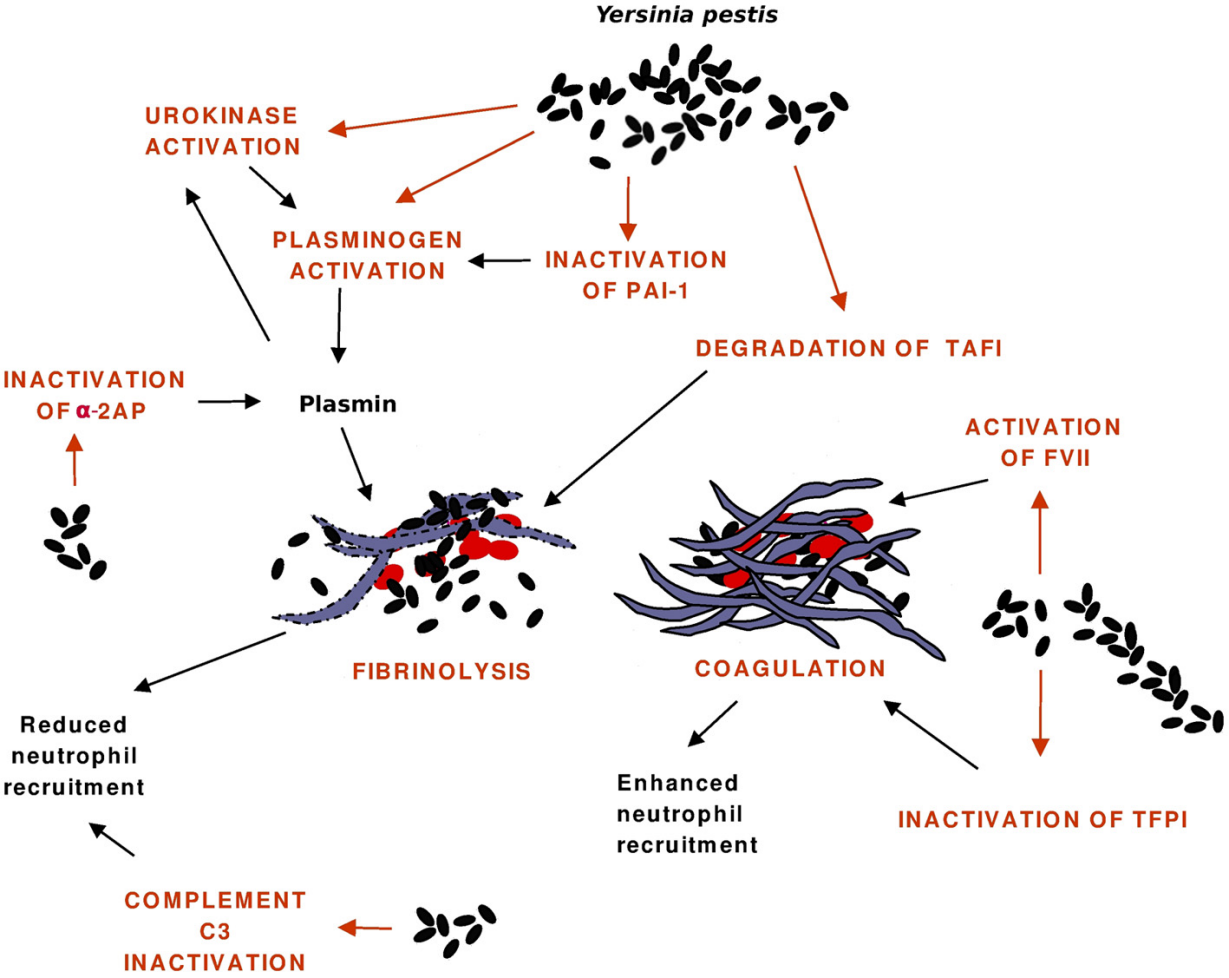
**Interactions of Pla with the hemostatic system.** Pla enhances fibrinolysis directly by activating plasminogen to plasmin and indirectly by inactivating the antiprotease plasminogen activator inhibitor-1 (PAI-1), by activating the precursor of the plasminogen activator urokinase, and by inactivating the plasmin inhibitor α_2_-antiplasmin (α_2_AP). Fibrin degradation allows bacterial dissemination and reduces engagement of neutrophils at the infection foci, which is also affected by the Pla-mediated cleavage of the C3 complement protein. Pla favors coagulation by activating the precursor of the enzyme Factor VII (FVII) and by inactivating the anticoagulant tissue factor pathway inhibitor (TFPI). Fibrin traps bacteria and decreases their dissemination, it also increases leukocyte engagement.

The hemostatic processes and reactions affected by Pla are shown in Figure 2, the evidence mainly comes from *in vitro* experiments. The first function observed for Pla was activation of human plasminogen, but recent research has shown that Pla has a broad activity on hemostatic processes and disrupts its control at various stages. Fibrinolysis is plasmin-mediated degradation of fibrin, and the system is composed of several proteins (serine proteases, their inhibitors, cellular receptors) that interact to regulate the generation of plasmin (reviewed by Vaughan and Declerck, 2003). The control occurs at the levels of protein-protein interactions and at the cellular level where the synthesis of fibrinolysis proteins are highly regulated.

### Activation of plasminogen

Plasminogen is an abundant circulating zymogen of plasmin synthesized primarily in the liver. Pla cleaves plasminogen at the same peptide bond R560-V561 as do the human physiological activators, uPA and the tissue-tPA, which yields the two-chain active enzyme plasmin (Sodeinde et al., 1992). The plasmin A chain contains lysine-binding domains (“kringles”) interacting with fibrin and with the plasmin inhibitor α_2_-antiplasmin (α_2_AP). Exposed lysines in fibrin or on cell surfaces and α_2_AP compete for binding to kringles, and therefore immobilization of plasmin on fibrin or on cell surfaces protects plasmin from inactivation by α_2_AP; lysine binding thus contributes to localization of plasmin proteolysis (Vaughan and Declerck, 2003). The B chain of plasmin contains the protease catalytic domain. Plasmin is a broad-spectrum serine protease that regulates fibrinolysis. It cleaves fibrin and fibrinogen at specific K-X and R-X bonds to produce small soluble degradation products (reviewed by Greenberg and Lai, 2003). Pla increases plasmin formation also indirectly by cleaving the zymogen form of uPA by a single, activating cut (Järvinen et al., 2013; see below).

Plasmin also enhances migration of phagocytic cells and metastatic tumor cells indirectly, through activation of latent metalloproteinases (MMPs) that degrade collagens, and also directly, by degrading laminin (Myöhänen and Vaheri, 2004; Schaller and Gerber, 2011). Thus, plasmin formation by Pla likely enhances migration of *Y. pestis* through main tissue barriers made up by inflammatory fibrin and basement membranes. Engagement of fibrin through α_M_β_2_ integrins on leukocyte surface is critical for leukocyte function (Flick et al., 2004), and another outcome of fibrin degradation or deficiency during bubonic plague is reduced activation of macrophages and neutrophils and lower local inflammatory response at the infection site (Degen et al., 2007). Fibrin also supports neutrophil-dependent T cell-mediated defense in mice against *Y. pestis* (Luo et al., 2013) Also, Pla degrades *in vitro* the complement protein C3, which should reduce trafficking of inflammatory cells to the infecton foci in plague (Sodeinde et al., 1992).

Infection studies in plasminogen-deficient and fibrinogen-deficient mice support the essential role of plasminogen as a Pla substrate. Although the general health of the plasminogen deficient mice is poor, they show increased resistance to *Y. pestis* infection, which underlines the role of plasmin formation in plague virulence (Degen et al., 2007). On the contrary, fibrinogen-deficient mice show increased sensitivity to infection which underlines the protective role of fibrin(ogen) in bubonic plague. In normal mice fibrin(ogen) deposition was reduced in liver lesions after Pla-positive *Y. pestis* infection (Degen et al., 2007). Also, expression of Pla during pneumonic plague associates with fibrin degradation (Lathem et al., 2007). Interestingly, Lathem et al. (2007) observed that deletion of *pla* dramatically decreases inflammatory cytokine response in intranasally infected mice, and concluded that Pla allows *Y. pestis* to cause lethal fulminant pneumonia in the lungs. It remains to be analysed whether this is a direct action of Pla protease on lung cells or an indirect effect resulting from increased cell numbers and translocation of type III secretion effector proteins into host cells (Akopyan et al., 2011). Pla could potentiate growth of *Y. pestis* in the lungs through cleavage of pulmonary antimicrobial peptides (Galvan et al., 2008). In this context, it is interesting that OmpT has been suggested to induce cytokine responses independently of the omptin-bound LPS (Brandenburg et al., 2005).

The *pla* gene is located in the pPCP1 virulence plasmid, and its predicted mature amino acid sequence is 100% identical in all pandemic *Y. pestis* branches. However, comparative genomics and substitutional analyses indicate that Pla has evolved since the gene was introduced into *Y. pestis*. The plague bacterium has diverged from *Y. pseudotuberculosis* which lacks *pla*, and the ancestral, non-pandemic lineages Microtus and Angola of *Y. pestis* carry Pla with I259, whereas the modern, pandemic lineages have T259 (Song et al., 2004; Haiko et al., 2009a; Eppinger et al., 2010). The change results from a single nucleotide substitution, and *in vitro* analyses showed that this substitution dramatically increases stability of the formed plasmin by preventing degradation of the B chain of plasmin by Pla (Haiko et al., 2009a). Pla in the modern lineages cleaves the plasminogen molecule at a single cut and is so far the only known omptin with T259 and with the ability to create stable plasmin activity. The others, such as PgtE of *S. enterica*, enable only transient plasmin activity as they also cleave the B chain, while others, such as OmpT of *E. coli*, cleave the plasminogen molecule poorly or not at all (Haiko et al., 2009b). This indicates that Pla has evolved toward creating stable plasmin activity and limited cleavage of the plasminogen molecule.

### Activation of scuPA

uPA is the physiological plasminogen activator associated with enhancement of cell migration and tissue remodeling, whereas tPA associates with fibrin degradation (Crippa, 2007; Schaller and Gerber, 2011). Synthesis and secretion of scuPA is upregulated in inflammatory environment by e.g., neutrophils recruited at the infection site, and it is secreted as a 411 amino acids-long zymogen that is processed to active uPA by plasmin and kallikrein. scuPA and uPA have receptors on several cell types, and uPA favors cell migration also by non-proteolytic processes (Montuori et al., 2013). Pla and several other omptins activate scuPA *in vitro* by a single cut at the peptide bond that is cleaved also by the physiological scuPA activator plasmin (Järvinen et al., 2013). PAI-1 binds to activated uPA and inhibits plasminogen activation, hence cleavage of PAI-1 by Pla (see below) may release this control and lead to uncontrolled plasmin formation by uPA.

### Inactivation of serine protease inhibitors

Serine protease inhibitors (serpins) are a major class of abundant protease inhibitors circulating in the human body and have an important role in controlling endogenous proteolysis and tissue remodeling. Serpins inhibit proteases by inserting their reactive center loop, which contains a bait peptide bond that mimics the normal target of the protease, into the catalytic groove of the target enzyme, where an irreversible covalent bond is rapidly formed and inactivates the enzyme. PAI-1 is present in a large variety of tissues and secreted by several human cells and a key controller of endogenous plasminogen activation by the serine proteases tPA and uPA (reviewed by Vaughan and Declerck, 2003; Schaller and Gerber, 2011). Pla cleaves the bait peptide bond R346-M347 in PAI-1 (Haiko et al., 2010) and thereby rapidly prevents its action to inhibit plasminogen activation. In circulation, most PAI-1 is bound to vitronectin which increases its stability; Pla degrades *in vitro* both PAI-1 and vitronectin present in the complex (Haiko et al., 2010). The circulating plasmin inhibitor α_2_AP is also inhibited and cleaved by Pla in a single, rapid cut (Kukkonen et al., 2001), and although not determined chemically, the cleavage most likely targets the bait peptide bond. Taken together, the serpinolytic activities of Pla disrupt the control of both plasminogen activation and plasmin activity, which then leads to uncontrolled proteolysis to advance bacterial migration and survival.

Besides inhibiting generation of the key enzyme plasmin and having a central role in maintaining normal hemostasis, PAI-1 has been implicated in processes such as wound healing, atherosclerosis, angiogenesis, and cell migration (Lijnen, 2005; van de Craen et al., 2012). Levels of PAI-1 are increased in infections by several Gram-negative pathogens, including *Y. pestis*, and are associated with unfavorable outcomes and enhanced mortality (Park et al., 1997; Zeeleder et al., 2006; Song et al., 2007; Comer et al., 2010; Kager et al., 2011; Lim et al., 2011). In accordance, infection models in PAI-1 knockout mice have shown that PAI-1 is protective in defense against sepsis caused by *Klebsiella pneumoniae, Burkholderia pseudomallei*, and *Y. enterocolitica* (Renckens et al., 2007; Hua et al., 2011; Kager et al., 2011; Luo et al., 2011) and lung infections by *Haemophilus influenzae* and *P. aeruginosa* (Goolaerts et al., 2011; Lim et al., 2011). These studies indicate that dampening the plasmin formation during acute inflammatory response helps the host to control bacterial infections, and contrariwise, inactivation of PAI-1 leads to low expression of pro-inflammatory cytokines and chemokines and low recruitment of leukocytes.

### Cleavage of thrombin-activatable fibrinolysis inhibitor

TAFI is an antifibrinolytic plasma protein that inhibits fibrinolysis by removing C-terminal lysines from fibrin. This reduces binding of plasminogen and tPA onto fibrin and thereby also impairs fibrinolysis. During coagulation, TAFI is activated to TAFIa, and the activation is mediated by several proteases that include thrombin, plasmin, trypsin, and neutrophil elastase (Kawamura et al., 2002; Marx et al., 2002). Pla cleaves TAFI near its C-terminus and so decreases thrombin-mediated activation of TAFI as well as antifibrinolytic potential of TAFIa (Valls Serón et al., 2010), which should favor plasmin action and fibrinolysis during infection. The role of TAFI in plague pathogenesis deserves further studies, in particular because TAFI has been found protective in septic yersiniosis (Luo et al., 2011).

## Pla enhances coagulation

TF is a transmembrane protein which under normal circumstances is expressed by e.g., epithelium of the skin, bronchus, and glomeruli but not by cells in contact with plasma (e.g., blood cells and endothelium of vessels) (reviewed by Broze, 2003). The expression of TF is induced in monocytes and endothelial cells by LPS, cytokines, and several other stimuli. In the event of injury to blood vessel wall, TF is exposed and complexes and activates the serine protease factor VII (FVII) to FVIIa. In a series of proteolytic activations, factors FIXa, and FXa and eventually the prothrombinase complex FXaFVa are formed (Sun, 2006). This complex cleaves prothrombin to thrombin, which then cleaves fibrinogen to fibrin and causes coagulation. Coagulation, fibrinolysis, and anticoagulation maintain a delicate physiological balance, and the main function of the anticoagulation system is to prevent or slow the propagation of fibrin clots. The major anticoagulants are antithrombin, protein C, and TFPI.

### Cleavage of TFPI

TFPI negatively regulates the coagulation system by inhibiting FXa and the TF-FVIIa complex. The endothelium is presumed to be the major source of TFPI *in vivo*, and a significant amount of TFPI remains membrane-associated. Unlike levels of PAI-1, the TFPI levels in plasma seem not to vary in response to e.g., pneumonia, and it was concluded that TFPI does not behave as an acute-phase reactant (Broze, 2003). On the other hand, TFPI is sensitive to inactivation by several host proteases (such as MMPs, elastase, thrombin, FXa, and plasmin), which are released from leukocytes or upregulated in coagulation, and TFPI inactivation is thus expected to increase during infection (Yun et al., 2009). Depletion of endogenous TFPI sensitizes rabbits to DIC induced by TF or LPS, stressing the importance of TFPI as an anticoagulant (Broze, 2003).

Yun et al. (2009) reported that Pla, as well as OmpT of *E. coli* and PgtE of *S. enterica*, cleave and inactivate TFPI; interestingly, as seen earlier with plasminogen as a substrate (Haiko et al., 2009a,b), the omptins show differing cleavage patterns of TFPI. Further, Pla, but not OmpT or PgtE, activated FVII to FVIIa, which is a major target for TFPI. The authors estimated that the inactivation rate of the naturally occurring, glycosylated TFPI by Pla is significantly higher than plasminogen activation by Pla and hence proposed that TFPI inactivation and fibrin formation protect *Y. pestis* during early stages of the infection. The estimation was, however, done before it was known that Pla inactivates PAI-1 and activates scuPA and hence probably underestimates fibrinolysis. The results however suggest that Pla accelerates initiation of coagulation by activating the first enzyme in blood-clotting (FVIIa) and inactivating its principal inhibitor in plasma (Yun et al., 2009). Fibrin augments mouse immune defense against *Y. pestis* (Luo et al., 2013). That efficient coagulation protects animals against plague is suggested by the interesting finding that population of prairie dogs in Arizona shows resistance to bubonic plague and has a ten-fold higher serum level of TF than the neighboring populations suffering from plague (Busch et al., 2011). The resistant prairie dog population also had a higher level of fibrinogen, and overall, immune system expression was different in the two populations.

## Conclusions

*Y. pestis* and Pla have a complex repertoire of interactions with the mammalian hemostatic system, and the observed *in vitro* and *in vivo* reactions favor both fibrinolysis and coagulation. A simple explanation would be that, once transmitted from the flea to the mammalian host, the hexa-acylated LPS molecules of *Y. pestis* cause a normal host response advancing coagulation. This could be strengthened by Pla-mediated cleavage of TFPI and activation of FVII and create a short-lived protective environment for the bacterium, as suggested by Yun and Morrissey (2009). Under these early conditions, the activity of Pla however is low because of the LPS structure and initiation of the coagulative cascade could be driven by the hexa-acylated LPS. Once cell multiplication at 37°C proceeds, the LPS is changed into the form that favors Pla activity and suppresses both Toll-like receptor 4-mediated immune response and fibrin-mediated leukocyte recruitment. This hypothesis emphasizes LPS alteration as a regulator of Pla activity and plague pathogenesis. Several biological aspects however remain unexplained. The activity of Pla in the bubonic and the pneumonic plague has somewhat opposite cellular effects. In bubonic plague, *Y. pestis* infiltrates lymph nodes without inducing a leukocyte reaction, which is in accordance with emerging fibrinolytic activity of Pla. In the lungs, to the contrary, Pla enables rapid replication of *Y. pestis* in the airways and is needed to cause a fulminant cytokine expression at the infection site. Transcriptomic analyses of coagulation-related genes in plague infection mostly detect changes after 2 or 3 days after the infection and have revealed increases in genes favoring both fibrinolysis and coagulation. This also reflects the complex and temporally diverse nature of the hemostatic system and the multiplicity of factors that are involved. It is likely that more host or *Y. pestis* factors targeting at Pla or targeted by Pla will be identified and have a role in directing Pla functions toward fibrinolysis or coagulation. An interesting recent example is given in the report that Pla proteolytically activates the YapE autotransporter of *Y. pestis* and *Y. pseudotuberculosis* that contributes to lymph node colonization during bubonic plague (Lawrence et al., 2013).

### Conflict of interest statement

The authors declare that the research was conducted in the absence of any commercial or financial relationships that could be construed as a potential conflict of interest.

## References

[B1] Agarkov A. Chauhan S. Lory P. J. Gilbertson S. R. Motin V. L. (2008). Substrate specificity and screening of the integral membrane protease Pla. Bioorg. Med. Chem. Lett. 18, 427–431 10.1016/j.bmcl.2007.09.104 17981463 PMC2263006

[B2] Akopyan K. Edgren T. Wang-Edgren H. Rosqvist R. Fahlgren A. Wolf-Watz H. (2011). Translocation of surface-localized effectors in type III secretion. Proc. Natl. Acad. Sci. U.S.A. 4, 1639–1644 10.1073/pnas.1013888108 21220342 PMC3029700

[B3] Beaufort N. Seweryn P. de Bentzmann S. Tang A. Kellermann J. Grebenchtchikov N. (2010). Activation of human pro-urokinase by unrelated proteases secreted by *Pseudomonas aeruginosa*. Biochem. J. 428, 473–482 10.1042/BJ20091806 20337595

[B4] Beaufort N. Wojciechowski P. Sommerhof C. P. Szmyd G. Dunin G. Eick S. (2008). The human fibrinolytic system is a target for the staphylococcal metalloprotease aureolysin. Biochem. J. 410, 157–165 10.1042/BJ20070650 17973626

[B5] Brandenburg K. Garidel P. Schromm A. B. Andra J. Kramer A. Egmond M. (2005). Investigation into the interaction of the bacterial protease OmpT with outer membrane lipids and biological activity of OmpT:lipopolysaccharide complexes. Eur. Biophys. J. 34, 28–41 10.1007/s00249-004-0422-3 15241571

[B6] Broze G. J. Jr. (2003) The tissue factor pathway of coagulation, in Thrombosis and Hemorrhage eds Loscalzo J. Schafer A. I. (Philadelphia, PA: Lippincott Williams and Wilkins), 62–80

[B7] Busch J. D. Van Andel R. Cordova J. Colman R. E. Keim P. Rocke T. E. (2011). Population dofference in host immune factors may influence survival of Gunnison's prairie dogs (*Cynomys gunnisoni*) during plague outbreaks. J. Wildl. Dis. 47, 968–973 22102668 10.7589/0090-3558-47.4.968

[B8] Caruso R. (1986). Fibrinogen/fibrin deposits in mesenteric lymphadenitis due to *Yersinia pseudotuberculosis* type I: morphologic, immunohistochemical and electron microscopic studies of one case. Basic Appl. Histochem. 30, 333–341 3539084

[B9] Caulfield A. J. Lathem W. W. (2012). Substrates of the plasminogen activator protease of *Yersinia pestis*. Adv. Exp. Med. Biol. 954, 253–260 10.1007/978-1-4614-3561-7_32 22782771 PMC3513919

[B10] Chromy B. A. Choi M. W. Murphy G. A. Gonzales A. D. Corzett C. H. Chang B. C. (2005). Proteomic characterization of *Yersinia pestis* virulence. J. Bacteriol. 187, 8172–8180 10.1128/JB.187.23.8172-8180.2005 16291690 PMC1291254

[B11] Chung M. C. Jörgensen S. C. Tonry J. H. Kaschanci E. Bailey C. Popov S. (2011). Secreted *Bacillus anthracis* taget the host fibrinolytic system. FEMS Immunol. Med. Microbiol. 62, 173–181 10.1111/j.1574-695X.2011.00798.x 21395696

[B12] Comer J. E. Sturdevant D. E. Carmody A. B. Virtaneva K. Gardner D. Long D. (2010). Transcriptomic and innate immune responses to *Yersinia pestis* in the lymph node during bubonic plague. Infect. Immun. 78, 5086–5098 10.1128/IAI.00256-10 20876291 PMC2981309

[B13] Crippa M. P. (2007). Urokinase-type plasminogen activator. Int. J. Biochem. Cell Biol. 39, 690–694 10.1016/j.biocel.2006.10.008 17118695

[B14] Degen J. L. Bugge T. H. Goguen J. D. (2007). Fibrin and fibrinolysis in infection and host defence. J. Thromb. Haemost. 5 Suppl. 1, 24–31 10.1111/j.1538-7836.2007.02519.x 17635705

[B15] Dekker N. Cox R. C. Kramer R. A. Egmond M. R. (2001). Substrate specificity of the integral membrane protease OmpT determined by spatially addressed peptide libraries. Biochemistry 40, 1694–1701 10.1021/bi0014195 11327829

[B16] Eppinger M. Worsham P. L. Nikolich M. P. Riley D. R. Sebastian Y. Mou S. (2010). Genome sequence of the deep-rooted *Yersinia pestis* strain Angola reveals new insight into the evolution and pangenome of the plague bacterium. J. Bacteriol. 192, 1685–1699 10.1128/JB.01518-09 20061468 PMC2832528

[B18] Eren E. Murphy M. Goguen J. van den Berg B. (2010). An active site network in the plasminogen activator Pla from *Yersinia pestis*. Structure 18, 809–818 10.1016/j.str.2010.03.013 20637417

[B17] Eren E. van den Berg B. (2012). Structural basis for activation of an integral membrane protease by lipopolysaccharide. J. Biol. Chem. 287, 23971–23976 10.1074/jbc.M112.376418 22645135 PMC3390672

[B19] Ferguson A. D. Welte W. Hofmann E. Lindner B. Holst O. Coulton J. W. (2000). A conserved structural motif for lipopolysaccharide recognition by prokaryotic and eukaryotic proteins. Structure 8, 585–592 10.1016/S0969-2126(00)00143-X 10873859

[B20] Fisher M. L. Castillo C. Mecsas J. (2007). Intranasal inoculation of mice with *Yersinia pseudotuberculosis* causes a lethal lung infection that is dependent on *Yersinia* outer proteins and PhoP. Infect. Immun. 75, 429–442 10.1128/IAI.01287-06 17074849 PMC1828392

[B21] Flick M. J. Du X. Witte D. P. Jirousková M. Soloviev D. A. Busuttil S. J. (2004). Leukocyte engagement of fibrin(ogen) via the integrin α_M_β_2_/MAC-1 receptor is critical for host inflammatory response *in vivo*. J. Clin. Invest. 113, 1596–1606 15173886 10.1172/JCI20741 PMC419487

[B22] Galvan E. M. Lasaro M. A. Schifferli D. M. (2008). Capsular antigen fraction 1 and Pla modulae the susceptibility of *Yersinia pestis* to pulmonary antimicrobial peptides such as cathelicidin. Infect. Immun. 76, 1456–1464 10.1128/IAI.01197-07 18227173 PMC2292867

[B23] Goolaerts A. Lafargue M. Song Y. Miyazawa B. Arjomandi M. Carlés M. (2011). PAI-1 is an essential component of the pulmonary response during *Pseudomonas aeruginosa* pneumoniae in mice. Thorax 66, 788–796 10.1136/thx.2010.155788 21768189 PMC3282176

[B24] Greenberg C. S. Lai T.-S. (2003). Fibrin formation and stabilization, in Thrombosis and Hemorrhage, eds Loscalzo J. Schafer A. I. (Philadelphia, PA: Lippincott Williams and Wilkins), 81–104

[B25] Guinet F. Avé P. Jones L. Huerre M. Carniel E. (2008). Defective innate cell response and lymph node infiltration specify *Yesinia pestis* infection. PLoS ONE 3:e1688 10.1371/journal.pone.0001688 18301765 PMC2244809

[B26] Haiko J. Kukkonen M. Ravantti J. J. Westerlund-Wikström B. Korhonen T. K. (2009a). The single substitution I259T, conserved in the plasminogen activator Pla of pandemic *Yersinia pestis* branches, enhances fibrinolytic activity. J. Bacteriol. 191, 4758–4766 10.1128/JB.00489-09 19465664 PMC2715710

[B27] Haiko J. Suomalainen M. Ojala T. Lähteenmäki K. Korhonen T. K. (2009b). Breaking barriers—attack on innate immune defences by omptin surface proteases of enterobacterial pathogens. Innate Immun. 15, 67–80 10.1177/1753425909102559 19318417

[B28] Haiko J. Laakkonen L. Juuti K. Kalkkinen N. Korhonen T. K. (2010). The omptins of *Yersinia pestis* and *Salmonella enterica* cleave the reactive center loop of plasminogen activator inhibitor 1. J. Bacteriol. 192, 4553–4561 10.1128/JB.00458-10 20639337 PMC2937412

[B29] Haiko J. Laakkonen L. Westerlund-Wikström B. Korhonen T. K. (2011). Molecular adaptation of a plant-bacterium outer membrane protease towards plague virulence factor Pla. BMC Evol. Biol. 11:43 10.1186/1471-2148-11-43 21310089 PMC3048539

[B30] Hua F. Ren W. Zhu L. (2011). Plasminogen activator inhibitor type-1 deficiency exaggerates LPS-induced acute lung injury through enhancing Toll-like receptor 4 signalling pathway. Blood Coagul. Fibrinolysis 22, 480–486 10.1097/MBC.0b013e328346ef56 21577093

[B31] Hwang B.-Y. Varadarajan N. Li H. Rodriguez S. Iverson B. L. Georgiou G. (2007). Substrate specificity of the *Escherichia coli* outer membrane protease OmpP. J. Bacteriol. 189, 522–530 10.1128/JB.01493-06 17085556 PMC1797397

[B32] Järvinen H. M. Laakkonen L. Haiko J. Johansson T. Juuti K. Suomalainen M. (2013). Human single-chain urokinase is activated by the omptins PgtE of *Salmonella enterica* and Pla of *Yersinia pestis* despite mutations of active site residues. Mol. Microbiol. [Epub ahead of print]. 10.1111/mmi.12293 23763588

[B33] Kager L. M. Wiersinga W. J. Roelofs T. H. Meijers J. C. M. Levi M. van't Veer C. (2011). Plasminogen activator inhibitor type 1 contributes to protective immunity during experimental Gram-negative sepsis (melioidosis). J. Thromb. Haemost. 9, 2020–2028 10.1111/j.1538-7836.2011.04473.x 21848642

[B34] Kawahara K. Tsukano H. Watanabe H. Lindner B. Matsuura M. (2002). Modification of the structure and activity of lipid A in *Yersinia pestis* lipopolysaccharide by growth temperature. Infect. Immun. 70, 4092–4098 10.1128/IAI.70.8.4092-4098.2002 12117916 PMC128165

[B35] Kawamura T. Okada N. Okada H. (2002). Elastase from activated human neutrophils activates procarboxypeptidase R. Microbiol. Immunol. 46, 225–230 12008933 10.1111/j.1348-0421.2002.tb02690.x

[B36] Knirel Y. A. Lindner B. Vinogradov E. V. Kocharova N. A. Senchenkova S. N. Shaikhutdinova R. Z. (2005). Temperature-dependent variations and intraspecies diversity of the structure of the lipopolysaccharide of *Yersinia pestis*. Biochemistry 44, 1731–1743 10.1021/bi048430f 15683257

[B37] Kramer R. A. Brandenburg K. Vandeputee-Rutten L. Werkhoven M. Gros P. Dekker N. (2002). Lipopolysaccharide regions involved in the activation of *Escherichia coli* outer membrane protease OmpT. Eur. J. Biochem. 269, 1746–1752 10.1046/j.1432-1327.2002.02820.x 11895445

[B38] Kramer R. A. Vandeputte-Rutten L. de Roon G. J. Gros P. Dekker N. Egmond M. R. (2001). Identification of essential acidic residues of outer membrane protease OmpT supports a novel active site. FEBS Lett. 505, 426–430 10.1016/S0014-5793(01)02863-0 11576541

[B39] Kramer R. A. Zandwijken D. Egmond M. R. Dekker N. (2000). *In vitro* folding, purification and characterization of *Escherichia coli* outer membrane protease OmpT. Eur. J. Biochem. 267, 885–893 10.1046/j.1432-1327.2000.01073.x 10651827

[B40] Kukkonen M. Lähteenmäki K. Suomalainen M. Kalkkinen N. Emödy L. Lång H. (2001). Protein regions important for plasminogen activation and inactivation of α_2_-antiplasmin in the surface protease Pla of *Yersinia pestis*. Mol. Microbiol. 40, 1097–1111 10.1046/j.1365-2958.2001.02451.x 11401715

[B41] Kukkonen M. Suomalainen M. Kyllönen P. Lähteenmäki K. Lång H. Virkola R. (2004). Lack of O-antigen is essential for plasminogen activation by *Yersinia pestis* and *Salmonella enterica*. Mol. Microbiol. 51, 215–225 10.1046/j.1365-2958.2003.03817.x 14651623

[B42] Lähteenmäki K. Edelman S. Korhonen T. K. (2005a). Bacterial metastasis: the host plasminogen system in bacterial infections. Trends Microbiol. 13, 79–85 10.1016/j.tim.2004.12.003 15680767

[B43] Lähteenmäki K. Kyllönen P. Partanen L. Korhonen T. K. (2005b). Antiprotease inactivation by *Salmonella enterica* released from infected macrophages. Cell. Microbiol. 7, 529–538 10.1111/j.1462-5822.2004.00483.x 15760453

[B44] Lathem W. W. Price P. A. Miller V. L. Goldman W. E. (2007). A plasminogen-activating protease specifically controls the development of primary pneumonic plague. Science 315, 509–513 10.1126/science.1137195 17255510

[B45] Lawrence M. B. Pennington J. Miller V. L. (2013). Acquisition of omptin reveals cryptic virulence function of autotransporter YapE in *Yersinia pestis*. Mol. Microbiol. 89, 276–287 10.1111/mmi.12273 23701256 PMC3708302

[B46] Lijnen H. R. (2005). Pleitropic functions of plasminogen activator inhibitor-1. J. Thromb. Haemost. 3, 35–45 10.1111/j.1538-7836.2004.00827.x 15634264

[B47] Lim J. H. Woo C. H. Li J.-D. (2011). Critical role of type 1 plasminogen activator inhibitor (PAI-1) in ealy host defence against nontypeable *Haemophilus influenzae* (NTHi) infection. Biochem. Biophys. Res. Commun. 414, 67–72 10.1016/j.bbrc.2011.09.023 21945446 PMC3360957

[B48] Liu H. Wang H. Qiu J. Wang X. Guo Z. Zhou D. (2009). Transcriptional profiling of a mice plague model: insight into interaction between *Yersinia pestis* and its host. J. Basic Microbiol. 49, 92–99 10.1002/jobm.200800027 18759226

[B49] Luo D. Lin J. S. Parent M. A. Mullarky-Kanevsky I. Szaba F. M. Kummer L. W. (2013). Fibrin facilitates both innate and T cell-mediated defense against *Yersinia pestis*. J. Immunol. 190, 4149–4161 10.4049/jimmunol.1203253 23487423 PMC3622124

[B50] Luo D. Szaba F. M. Kummer L. W. Plow E. F. Mackman N. Gailani D. (2011). Protective roles for fibrin, tissue factor, plasminogen activator inhibitor-1, and thrombin activable fibrinolysis inhibitor, but not factor XI, during defence against the Gram-negative bacterium *Yersinia enterocolitica*. J. Immunol. 187, 1866–1876 10.4049/jimmunol.1101094 21724997 PMC3150340

[B51] Marx P. F. Dawson P. E. Bouma B. N. Meijers J. C. (2002). Plasmin-mediated activation and inactivation of thrombin-activable fibrinolysis inhibitor. Biochemistry 41, 6688–6696 10.1021/bi015982e 12022872

[B52] McCarter J. D. Stephens D. Shoemaker K. Rosenberg S. Kirsch J. F. Georgiou G. (2004). Substrate specificity of the *Escherichia coli* outer membrane protease OmpT. J. Bacteriol. 186, 5919–5925 10.1128/JB.186.17.5919-5925.2004 15317797 PMC516829

[B53] Montminy S. W. Khan N. McGrath S. Walkowicz M. J. Sharp F. Conlon J. E. (2006). Virulence factors of *Yersinia pestis* are overcome by a strong lipopolysaccharide response. Nat. Immunol. 7, 1066–1073 10.1038/ni1386 16980981

[B54] Montuori N. Cosimato V. Rinaldi L. Rea V. E. A. Alfano D. Ragno P. (2013). uPAR regulates pericellular proteolysis through a mechanism involving integrins and fMLF-receptors. Thromb. Haemost. 31, 309–318 23238745 10.1160/TH12-08-0546

[B55] Motin V. L. Georgescu A. M. Fitch J. P. Gu P. P. Nelson D. O. Mabery S. L. (2004). Temporal global changes in gene expression during temperature transition in *Yesinia pestis.* J. Bacteriol. 186, 6298–6305 10.1128/JB.186.18.6298-6305.2004 15342600 PMC515171

[B56] Myöhänen H. Vaheri A. (2004). Regulations and interactions in the activation of cell-associated plasminogen. Cell. Mol. Life Sci. 61, 2840–2858 10.1007/s00018-004-4230-9 15558213 PMC11924493

[B57] Park C.-G. Goh Y.-J. Kook S.-H. Park H.-K. Kim B.-J. Cha C.-Y. (1997). Incresed expression of urokinase type plasminogen activator (u-PA), plasminogen activator inhibitor-1 (PAI-1), and collagenases in Caco-2 cells infected by *Salmonella typhimurium*. JKMS 12, 23–31 9142656 10.3346/jkms.1997.12.1.23 PMC3054269

[B58] Prior J. L. Parkhill J. Hitchen P. G. Mungall K. L. Baker S. G. Stevens K. (2001). The failure of different strains of *Yersinia pestis* toproduce lipopolysaccharide O-antigen under different growth conditions is due to mutations in the O-antigen gene cluster. FEMS Microbiol. Lett. 197, 229–233 10.1111/j.1574-6968.2001.tb10608.x 11313139

[B59] Ramu P. Lobo L. A. Kukkonen M. Bjur E. Suomalainen M. Raukola H. (2008). Activation of pro-matrix metalloproteinase-9 and degradation of gelatine by the surface protease PgtE of *Salmonella enterica* serovar Typhimurium. Int. J. Med. Microbiol. 298, 263–278 10.1016/j.ijmm.2007.06.004 17888724

[B60] Renckens R. Roelefs J. J. Bonta P. I. Florquin S. de Vries C. J. Levi M. (2007). Plasminogen activator inhibitor type 1 is protective during severe Gram-negative pneumoniae. Blood 109, 1593–1601 10.1182/blood-2006-05-025197 17032919

[B61] Schaller J. Gerber S. S. (2011). The plasmin-antiplasmin system: structural and functional aspects. Cell. Mol. Life Sci. 68, 785–801 10.1007/s00018-010-0566-5 21136135 PMC11115092

[B62] Sebbane F. Lemaitre N. Sturdevant D. E. Rebeil R. Virtaneva K. Porcella S. F. (2006). Adaptive response of *Yersinia pestis* to extracellular effectors of innate immunity during bubonic plague. Proc. Natl. Acad. sci. U.S.A. 103, 11766–11771 10.1073/pnas.0601182103 16864791 PMC1518801

[B63] Semeraro N. Ammollo C. T. Semeraro F. Colucci M. (2012). Sepsis, thrombosis and organ dysfunction. Thromb. Res. 129, 290–295 10.1016/j.thromres.2011.10.013 22061311

[B64] Skurnik M. Peippo A. Ervelä E. (2000). Characterization of the O-antigen gene clusters of *Yersinia pseudotuberculosis* and the cryptic O-antigen gene cluster of *Yersinia pestis* shows that the plague bacillus is most closely related to and has evolved from *Y. pseudotuberculosis* serotype O:1b. Mol. Microbiol. 37, 316–330 10.1046/j.1365-2958.2000.01993.x 10931327

[B65] Sodeinde O. A. Subrahmanyan Y. V. Stark K. Quan T. Bao Y. Goguen J. D. (1992). A surface protease and the invasive character of plague. Science 258, 1004–1007 10.1126/science.1439793 1439793

[B66] Song Y. Lynch S. V. Flanagan J. Zhuo H. Tom W. Dotson R. H. (2007). Increased plasminogen activator inhibitor-1 concentrations in bronchoalveolar lavage fluids are associated with increased mortality in a cohort of patients with *Pseudomonas aeruginosa*. Anesthesiology 106, 252–261 10.1097/00000542-200702000-00012 17264718

[B67] Song Y. Tong Z. Wang J. Wang L. Guo Z. Han Y. (2004). Complete genome sequence of *Yersinia pestis* strain 91001, an isolate avirulent to humans. DNA Res. 11, 179–197 10.1093/dnares/11.3.179 15368893

[B68] Sun H. (2006). The interaction between pathogens and the host coagulation system. Physiology 21, 281–288 10.1152/physiol.00059.2005 16868317

[B69] Suomalainen M. Lobo L. A. Brandenburg K. Lindner K. Virkola R. Knirel Y. A. (2010). Temperature-induced changes in the lipopolysaccharide of *Yersinia pestis* affect plasminogen activation by the Pla surface protease. Infect. Immun. 78, 2644–2652 10.1128/IAI.01329-09 20368351 PMC2876559

[B72] Valls Serón M. Haiko J. Korhonen T. K. de Groot P. G. Meijers C. M. M. (2010). Thrombin-activable fibrinolysis inhibitor is degraded by *Salmonella enterica* and *Yersinia pestis*. J. Thromb. Haemost. 8, 2232–2240 10.1111/j.1538-7836.2010.04014.x 20704647

[B73] van de Craen B. Declerck P. J. Gils A. (2012). The biochemistry, physiology and pathological roles of PAI-1 and the requirements for PAI-1 inhibition *in vivo*. Thromb. Res. 130, 576–585 10.1016/j.thromres.2012.06.023 22801256

[B75] Vandeputte-Rutten L. Kramer R. A. Kroon J. Dekker N. Egmond M. R. Gros P. (2001). Crystal structure of the outer membrane protease OmpT from *Escherichia coli* suggests a novel catalytic site. EMBO J. 20, 5033–5039 10.1093/emboj/20.18.5033 11566868 PMC125623

[B74] van Gorp E. C. M. Suhart C. ten Cate H. Dolmans W. M. V. van der Meer J. W. M. ten Cate J. W. (1999). Review: Infectious diseases and coagulation disorders. J. Inf. Dis. 180, 176–186 10.1086/314829 10353876

[B76] Vaughan D. E. Declerck P. J. (2003). Regulation of fibrinolysis, in Thrombosis and Hemorrhage, eds Loscalzo J. Schafer A. I. (Philadelphia, PA: Lippincott Williams and Wilkins), 105–119

[B70] Yun T. H. Cott J. E. Tapping R. I. Slauch J. M. Morrissey J. H. (2009). Proteolytic inactivation of tissue factor pathway inhibitor by bacterial omptins. Blood 113, 1139–1148 10.1182/blood-2008-05-157180 18988866 PMC2635079

[B71] Yun T. H. Morrissey J. H. (2009). Polyphosphate and omptins: novel bacterial procoagulant agents. J. Cell. Mol. Med. 13, 4146–4153 10.1111/j.1582-4934.2009.00884.x 19725923 PMC2891932

[B77] Zeeleder S. Schroeder V. Hack C. E. Kohler H. P. Wuillemin W. A. (2006). TAFI and PAI-1 levels in human sepsis. Thromb Res. 118, 205–212 10.1016/j.thromres.2005.06.007 16009400

